# Acute induction of anxiety in humans by delta-9-tetrahydrocannabinol related to amygdalar cannabinoid-1 (CB1) receptors

**DOI:** 10.1038/s41598-017-14203-4

**Published:** 2017-11-03

**Authors:** Sagnik Bhattacharyya, Alice Egerton, Euitae Kim, Lula Rosso, Daniela Riano Barros, Alexander Hammers, Michael Brammer, Federico E. Turkheimer, Oliver D. Howes, Philip McGuire

**Affiliations:** 1 Department of Psychosis Studies, King’s College London, Institute of Psychiatry, Psychology & Neuroscience, De Crespigny Park, London, SE5 8AF UK; 2 Department of Psychiatry, Seoul National University College of Medicine, Seoul, Republic of Korea; 3 Department of Neuroscience, Imperial College London, London, UK; 4 King’s College London & Guy’s and St Thomas’ PET Centre, School of Biomedical Engineering and Imaging Sciences, Faculty of Life Sciences and Medicine, King’s College London, 4th floor Lambeth Wing, St Thomas’ Hospital, Westminster Bridge Road, London, SE1 7EH UK; 5 Department of Neuroimaging, Centre for Neuroimaging Sciences, PO Box 089, King’s College London, Institute of Psychiatry, Psychology & Neuroscience, De Crespigny Park, London, SE5 8AF UK; 6 Medical Research Council Clinical Sciences Centre, Hammersmith Hospital, London, UK

## Abstract

Use of Cannabis, the most widely used illicit drug worldwide, is associated with acute anxiety, and anxiety disorders following regular use. The precise neural and receptor basis of these effects have not been tested in man. Employing a combination of functional MRI (fMRI) and positron emission tomography (PET), we investigated whether the effects of delta-9-tetrahydrocannabinol (delta-9-THC), the main psychoactive ingredient of cannabis, on anxiety and on amygdala response while processing fearful stimuli were related to local availability of its main central molecular target, cannabinoid-1 (CB1) receptors in man. Fourteen healthy males were studied with fMRI twice, one month apart, following an oral dose of either delta-9-THC (10 mg) or placebo, while they performed a fear-processing task. Baseline availability of the CB1 receptor was studied using PET with [^11^C]MePPEP, a CB1 inverse agonist radioligand. Relative to the placebo condition, delta-9-THC induced anxiety and modulated right amygdala activation while processing fear. Both these effects were positively correlated with CB1 receptor availability in the right amygdala. These results suggest that the acute effects of cannabis on anxiety in males are mediated by the modulation of amygdalar function by delta-9-THC and the extent of these effects are related to local availability of CB1 receptors.

## Introduction

Cannabis is the world’s most commonly used illicit substance^1^. Although most recreational users smoke cannabis for its relaxing effects, the drug has a range of adverse effects including the induction of acute anxiety^2–5^ and, development of anxiety disorders in regular users^6^. While these effects of cannabis are mediated by its main psychoactive constituent, delta-9-tetrahydrocannabinol (delta-9-THC)^3^, the extract of the cannabis plant has many other ingredients, some of which e.g. Cannabidiol may have anxiolytic effects^3^. Consistent with this, and evidence that many of the behavioural effects of exogenous cannabinoids can be linked to their effects on the endocannabinoid system^7^, a large body of preclinical evidence has accumulated implicating different components of the endocannabinoid system in emotional processing and anxiety^8^. Preclinical studies suggest that contradictory evidence regarding the precise effect of exogenous cannabinoids on anxiety, in terms of an anxiolytic or anxiogenic response, may be linked to dose of the cannabinoid^7^ as well as its site of action^9^, while there is less mechanistic detail from human studies^8^. Preclinical evidence also show that delta-9-THC is a partial agonist at cannabinoid-1 (CB1) receptors, which is also the main target of endocannabinoids in the brain^8^ consistent with human evidence that a single dose of CB1 receptor antagonist Rimonabant is able to block the acute subjective intoxication caused by smoked cannabis^10^. However, whether the effects of delta-9-THC are related to the availability of CB1 receptors in the brain has not yet been demonstrated in humans.

We recently reported that the anxiogenic effects of delta-9-THC in humans were related to its effect on neural activity in the amygdala^3^, a region that plays a critical role in mediating anxiety^11^ and is rich in CB1 receptors^12^. In contrast, another study^13^ reported an attenuating effect of a smaller dose of delta-9-THC on amygdala function whilst processing social signals of threat. While inconsistent in terms of the precise direction of effect, results of both these studies were consistent with evidence regarding the biphasic nature of the effects of delta-9-THC on anxiety^7^, and highlighted the central role of amygdala as the neural substrate mediating the effects of delta-9-THC on emotional processing in man. Using neuroanatomically localized microinjection of drugs, studies in freely moving rodents have linked anxiogenic effects of delta-9-THC to a specific CB1 receptor-dependent effect in the amygdala, while anxiolytic effects have been linked to its effects in the prefrontal cortex or hippocampus^9^. However, it is unclear whether the effects of delta-9-THC on anxiety and on amygdala function during the processing of fearful stimuli are related to local availability of the CB1 receptor in humans.

Hence, in the present study, we investigated whether the acute effects of delta-9-THC on anxiety and on amygdala function as measured using fMRI during the processing of fear were related to availability of CB1 receptors in the amygdala in an independent sample of healthy male volunteers, with minimal previous cannabis and other illicit drug use. CB1 receptor availability was studied using PET with [^11^C]MePPEP, a CB1-selective, inverse agonist radioligand with high and stable brain uptake *in vivo*, high selectivity and specificity for CB1 receptors and good-to-excellent reproducibility on test-retest scans^14–18^. Our first hypothesis was to confirm that acute administration of delta-9-THC would be associated with increased anxiety and engagement of amygdala during the processing of fearful stimuli. Our second hypothesis, which was the main focus of the present study, was that magnitude of the effects of delta-9-THC on anxiety and on amygdalar activation would be related to the local availability of CB1 receptors. We specifically predicted that there would be a direct correlation between baseline CB1 availability in the amygdala and effect of delta-9-THC on anxiety and amygdala response during the processing of fear, with greater CB1 availability being linked to greater effect of delta-9-THC on anxiety and amygdala activity.

## Materials and Methods

**Figure 1:**
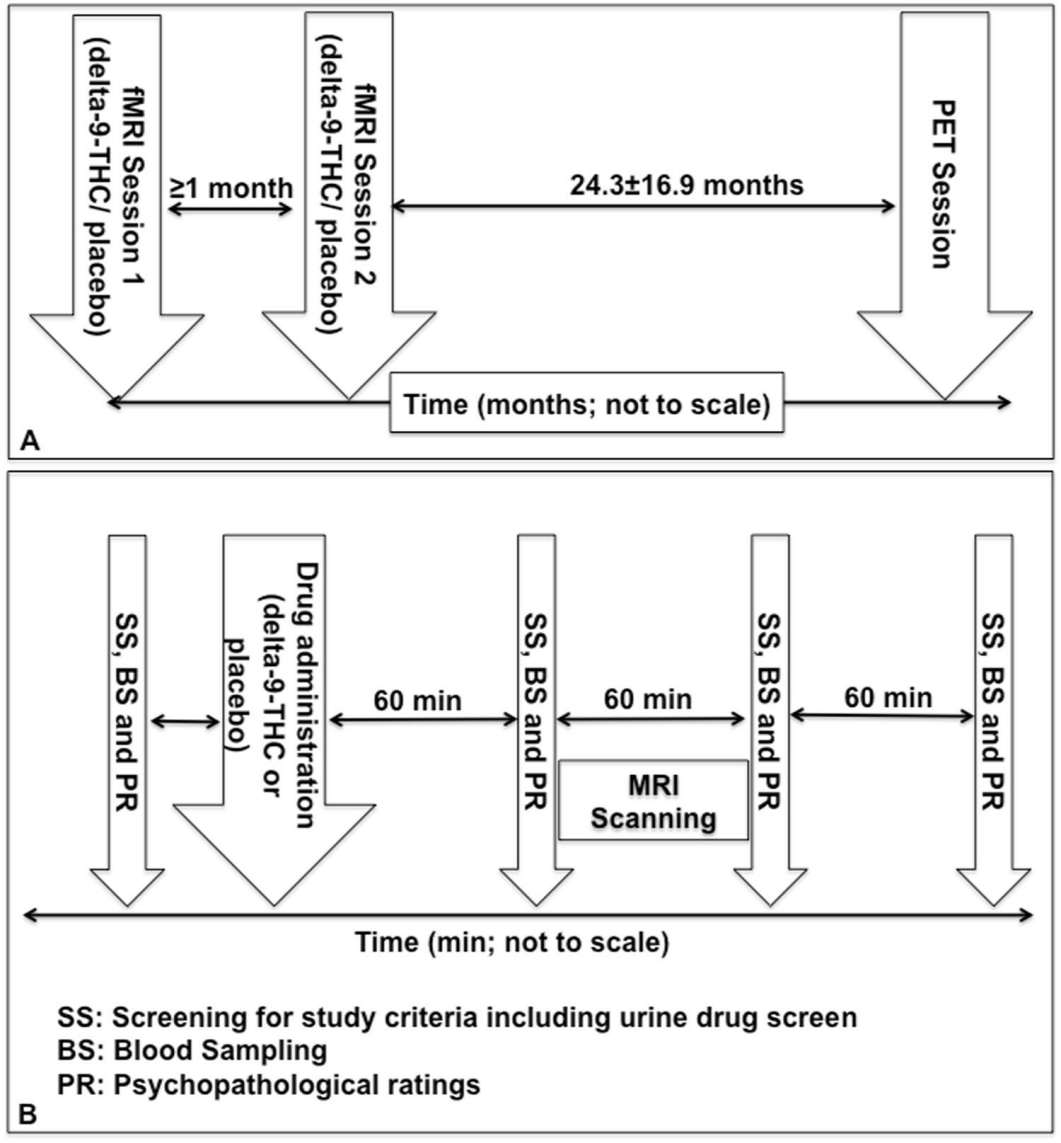
Overall experimental timeline for fMRI and PET sessions (**A**) and experimental schedule for fMRI sessions (**B**).

This study was conducted in accordance with the Declaration of Helsinki after obtaining approval from the Joint South London and Maudsley and Institute of Psychiatry research ethics committee and radiation protection agency (ARSAC). After complete description of the study to subjects, written informed consent was obtained. Participants were studied using two imaging techniques [functional magnetic resonance imaging (fMRI) and positron emission tomography (PET)] on separate occasions (Fig. 1). All participants completed all components of the fMRI scanning involving acute pharmacological challenge first followed by PET scanning.

### Subjects

Out of a total of twenty-one individuals who were approached, fourteen right-handed, English-speaking, healthy males (mean ± SD age of 23.79 ± 4.45 years and NART^19^ IQ of 98.15 ± 5.01), without a personal or family history of psychiatric illness completed the study. They were screened for a personal history of psychiatric illness with a structured diagnostic interview^20^. Illicit substance use including cannabis use was assessed using the Addiction severity index and abuse was defined as “moderate use of small quantities regularly or large amounts occasionally”^21^. All subjects had used cannabis at least once, but less than 25 times in their lifetime (Table 1). None of them used more than 21 units/week of alcohol or other illicit drugs on a regular basis or satisfied criteria for alcohol or substance abuse or dependence. None had used cannabis or other illicit drugs in the 3 months before the study and all were asked to abstain from all recreational drug use for the duration of the study. Only five had ever smoked cigarettes, and none had ever smoked more than two cigarettes/day. None of the participants satisfied criteria suggestive of nicotine dependence or abuse. As they only described ever smoking a maximum of 2 cigarettes per day and agreed to abstain from smoking on the day of scanning, we did not carry out breathalyser analyses of carbon monoxide levels on either the PET or MRI scanning days.

**Table 1:** Psychoactive substances used by study participants.

Lifetime Illicit drug use	
Cannabis	<5 times: 2 subjects;
	5–20 times: 11 subjects;
	25 times: 1 subject
Amphetamines	No use
LSD/Psilocybin	4 subjects ‡ (all had experimented a few times)
Cocaine	No use
Opiate	1 subject (experimented a few times)
MDMA	4 subjects (all of them had experimented a few times)
**Other psychoactive substances (current use)**	
Nicotine	5 subjects ever smoked cigarettes;
	Mean number of cigarettes smoked/day- 0.46 (SD-0.69) (range 0–2/day);
Caffeine	13 subjects;
	Mean number of cups of coffee, tea or caffeinated drinks/day- 1.77 (SD-1.32) (range 0–4)

‡ 1 subject had experimented with both opiates & Psilocybin.

### Functional Magnetic Resonance Imaging Scans

#### Design

We employed an established, double-blind, placebo-controlled, repeated-measures, within-subject, crossover design that we have employed before^3,22–24^ to compare the effects of orally administered 10 mg of delta-9-THC (approximately 99.6% pure, THC-Pharm, Frankfurt, Germany) with matched placebo capsules (Fig. 1). This dose of delta-9-THC was chosen as it is within the range used recreationally^25^ and previous work suggested that the dose is sufficient to produce symptomatic and neural effects without the symptomatic effects being severe enough to affect compliance with study procedures^22,26^. It is well-known that delta-9-THC has a dose-dependent effect on anxiety in man, with lower doses generally having an anxiolytic effect and oral doses of 10 mg or higher having an anxiogenic effect^3,7,13,25^. As the focus of the study was to investigate the neural mechanisms underlying the symptomatic/cognitive effects of cannabis, we decided to employ a dose delta-9-THC that would induce such effects without the effects being severe enough to interfere with participation in the study. In the present study, we employed the oral route of administration because it produces consistent and reliable effects^25^ allowing a more sustained dose of delta-9-THC than the inhaled route, which results in faster onset and more severe but shorter lasting effects^27^. Functional neuroimaging studies suggest that the effects of cannabis in the brain are comparable between studies that employ various routes of administration^28^.

Participants were tested on two occasions separated by at least one month. The order of drug administration was pseudo-randomized across subjects, so that an equal number received each of the treatment conditions during the first or second session. On the day of each session, subjects were asked to have a light standardized breakfast after an overnight fast and advised to get at least six hours sleep the night before. They were asked to refrain from smoking for 4 hours (if relevant), caffeine for 12 hours and alcohol for 24 hours. All subjects had a negative urine drug screen for opiates, cocaine, amphetamines, benzodiazepines and delta-9-THC on testing before each session using immunometric assay kits. Illicit substance use was assessed using a structured clinical interview and the Addiction severity index^21^. Psychopathological ratings (which included level of intoxication, anxiety, positive and negative psychotic symptoms) and venous blood samples (using an indwelling intravenous catheter inserted into a subcutaneous vein in the forearm of the non-dominant arm) were obtained immediately before, and at 1, 2 and 3 hours post drug administration. Anxiety was measured using the Spielberger state-trait anxiety inventory (STAI-S)^29^, positive and negative psychotic symptoms were assessed by an experienced clinician using the Positive and Negative Syndrome Scale (PANSS)^30^, while subjective intoxication was measured using the self-rated Analogue Intoxication scale (AIS)^31^.

MRI scans were acquired between 1 and 2 hours after drug administration, as previous work indicated that an identical dose of orally administered delta-9-THC produced sustained blood levels over this period^2^, and that the neural and behavioral effects of delta-9-THC during the emotional (fear) processing task, and its symptomatic effects were evident within this period^3^. Except when MR scanning was performed, subjects remained seated in a quiet room throughout the session.

#### Cognitive task inside the MRI scanner

The emotional (fear) processing task has been described in detail elsewhere^3,23^. The blood oxygen level-dependent (BOLD) haemodynamic response was measured while subjects viewed fearful faces, which were contrasted with faces with neutral expressions, in an event-related design. Over a 6-minute period, participants viewed 60 facial stimuli in total (30 different facial stimuli each presented twice) presented for 2-seconds each, from a list of 10 different facial identities. The order of presentation of facial identities and expression type was pseudo-randomized, such that the same identity or expression type was not presented in successive trials. Subjects viewed a fixation cross during the inter-stimulus interval, which was varied from 3–8 seconds according to a Poisson distribution, with an average interval of 5.9 seconds. They were asked to indicate the gender of the face by pressing one of two buttons. The speed and accuracy of responses was recorded on-line throughout image acquisition. Autonomic arousal related to the presentation of fearful faces was recorded on-line throughout image acquisition by measuring the electrodermal response (described below).

#### Image acquisition

All MR images were acquired on a 1.5 Tesla Signa system (GE) at the Institute of Psychiatry, London. T2*-weighted images were acquired with echo time (TE) of 40 msec, flip angle 90°, in 16 axial planes (7 mm thick), parallel to the anterior commissure-posterior commissure line. The emotional (fear) processing task was studied using a TR of 2000 msec and TE of 40 msec. To facilitate anatomical localization of activation, a high-resolution inversion recovery image dataset was also acquired, with 3mm contiguous slices and an in-plane resolution of 3 mm (TR 16000 ms, TI 180 ms, TE 80 ms).

#### Image analysis

fMRI data were analysed using XBAMv4 (http://www.kcl.ac.uk/ioppn/depts/neuroimaging/research/imaginganalysis/Software/XBAM.aspx), employing a non-parametric approach that we have used previously with the same paradigm^3^. This method does not assume a Gaussian population distribution, as such an assumption is very difficult to test in small groups with neuroimaging data and when tested, is often found to be violated^32,33^. Instead, this method uses median statistics to control for outlier-effects and employs permutation rather than normal theory-based inference^34^. According to this method, the test statistic is computed by standardizing for individual differences in residual noise before embarking on a second-level, multi-subject testing, using robust permutation-based methods, employing a mixed-effects approach. Data from all subjects met criteria for image quality and movement (<1 mm displacement in any one direction) and were included for analysis. Images were realigned to correct for head motion^35^ and smoothed with a Gaussian filter (full width at half maximum 7.2 mm). Individual activation maps were created by convolving the experimental design with two gamma variate functions to model the blood oxygen level-dependent (BOLD) response. The best fit between the weighted sum of these convolutions and the time series at each voxel was computed using the constrained BOLD effects model^36^. Following a least squares fitting of this model, the sum of squares ratio (SSQ) was estimated at each voxel, followed by permutation testing to determine significantly activated voxels specific to each condition^37^. The detection of activated voxels was then extended from voxel to cluster level^35^. SSQ ratio maps for each individual were transformed into standard stereotactic space^38^ using a two-stage warping procedure^32^. Group activation maps were computed for each treatment condition by determining the median SSQ ratio at each voxel. These maps were compared using nonparametric-repeated measures ANOVA, with a voxel-wise threshold of *p* = 0.05 and the cluster-wise threshold set such that the total number of false positive clusters per brain volume was <1: the *p* value at which the latter occurred is quoted. Only areas of activation that survived this correction threshold are reported.

The principal advantages of cluster-level testing are that it confers greater sensitivity by incorporating information from more than one voxel in the test statistic and also substantially reduces the search volume or number of tests required for a whole-brain analysis, thereby mitigating the multiple comparisons problem.

For each treatment condition (delta-9-THC and placebo), we contrasted the active task (fearful faces) condition against the baseline (neutral faces) condition to identify the brain regions engaged by the processing of fear. Employing a whole-brain analysis approach, we then carried out nonparametric repeated-measures ANOVA to identify brain regions that were activated by delta-9-THC relative to the placebo condition.

#### Electrodermal Activity (Skin Conductance response)

Skin conductance response (SCR) was recorded during the fMRI scanning while performing the emotional (fear) processing task, via a pair of silver-silver chloride electrodes with 0.05 M sodium chloride gel placed on the distal phalanges of 2^nd^ and 3^rd^ digits of the nondominant hand. The electrode pairs were supplied with a constant voltage and the change in current representing conductance was recorded using the DC amplifier. The number, amplitude and rise-time of SCRs fluctuations were recorded. A fluctuation was defined by an unambiguous increase (0.01 microS) with respect to each pre-target stimulus baseline and occurring 0.5–3 sec after the target face stimulus^39^. The fluctuation amplitude was measured as the difference in skin conductance (SC) level from the onset (the SC measure before the first rising data point) to the fluctuation peak. The number and amplitude of SCRs were scored using customized software that allows each SCR to be linked to the individual eliciting stimulus. Usable SCR data were available from 11 participants.

### PET Scanning

Mean interval between PET and fMRI scan acquisition was 25.3 ± 16.9 months. There was no significant relationship between inter-scan interval and CB1 receptor availability in the right amygdala (r = 0.017, *p* = 0.95).

#### Radiochemistry

[^11^C]MePPEP was synthesized on site by Hammersmith Imanet using a procedure described previously^14,40^.

#### [^11^*C]MePPEP imaging*

Participants were asked to abstain from cigarettes, alcohol or other substance use for at least 12 hours (and at least three months in the case of cannabis) prior to PET imaging. All participants had a negative urine drug screen for opiates, cocaine, amphetamines, benzodiazepines and delta-9-THC on testing immediately before the scan using immunometric assay kits.

[^11^C]MePPEP PET scans were acquired on an ECAT HR + 962 PET scanner (CTI/Siemens) in 3D mode, with an axial field of view of 15.5 cm. A 10-minute transmission scan was performed using a rotating 137Cs point source prior to radiotracer injection to correct for attenuation and scatter. Approximately 370 MBq of [^11^C]MePPEP was administered by intravenous injection 30 seconds after the start of the PET imaging as a smooth bolus over 15 seconds. The dose [Mean (SD)] of MePPEP injected was 361.79 (14.6) MBq, and the specific activity [Mean (SD)] was 48.49 (14.5) GBq/µmol. Emission data were acquired in list mode for 95 minutes, and rebinned into 35 time-frames (comprising a 30-second background frame, six 10-second frames, three 20-second frames, three 30-second frames, four 60-second frames, six 120-second frames, nine 300-second frames, and three 600-second frames). Positioning laser was used to monitor and maintain head position during the scanning. If movement was noticed, participants were repositioned and underwent a second transmission scan at the end of the dynamic scan. Post-hoc frame-by-frame realignment method as described below was used to compensate for head movement during the dynamic scan.

PET data were reconstructed using the reprojection algorithm^41^ employing a two-stage procedure: initially a first-pass image from the subset of direct projections corresponding to the complete set of projections acquired in 2D was reconstructed using the 2D filtered back projection algorithm (FBP) and a ramp filter (kernel 2mm full width at half maximum) cutoff at Nyquist frequency; this image was used to forward project the missing projections in 3D. The 3D FBP algorithm was then used to reconstruct a second-pass image from the merged set of measured and estimated projections in 3D. For the extension of the 2D FBP algorithm to one more dimension, the ramp filter was replaced by the Colsher filter, also cutoff at Nyquist frequency. Measured attenuation maps were segmented^42^ and used for attenuation and model-based scatter correction^43^. Reconstructed voxel sizes were 2.092 mm × 2.092 mm × 2.42 mm and the final spatial resolution of reconstructed images was about 6 mm × 6 mm × 5 mm full width at half maximum.

#### Derivation of input function

Arterial plasma input fractions were derived using an approach described before^44^. Prior to the scan, a 22-gauge cannula was inserted into a radial artery after local anaesthesia with 0.5% bupivacaine. During the first 15 minute, arterial blood was continuously withdrawn at a sampling rate of 5 ml/min and measured in a BGO detection system as described previously^45,46^. To quantify plasma and whole blood radioactivity, as well as to allow quantification of the parent fraction of the radiotracer, intermittent discrete samples (10 ml) were taken with heparinized syringes before the scan and at the following time points after the start of scanning: 3, 5, 10, 15, 20, 30, and 50 min. A larger sample (17 ml) was taken at 75 min to allow quantification despite radioactive decay. Quantification of parent fraction was not possible at 90 min and only 3 ml was withdrawn at this point for plasma and whole blood radioactivity measurement. For the generation of the plasma input functions, the time course of the plasma to blood ratio obtained from the first six discrete arterial samples at 3, 5, 10, 15, 20 and 30 min scan times were fitted to a sigmoidal function with four free parameters. Then arterial whole blood activities recorded by the continuous detector system^46^ were corrected to obtain a plasma activity curve for the first 15 minutes of the scan. This curve was then combined with the discrete plasma activity measurements at 20, 30, 50, 60 and 75 min to generate an input function describing the plasma activity throughout the entire scan. An input function of the activity concentration due to unmetabolised [^11^C]MePPEP in plasma was then created by multiplying the total plasma activity input function with the function obtained from the fit of a sigmoidal model for the parent function in plasma to the eight measurements of the parent compound during the scan as described before^40,45,47^. Finally, the time delay of the arrival of the radioactivity bolus at the peripheral sampling site relative to the brain site was determined^48^. All calculations were performed using Matlab (The Mathworks Inc., Natick, MA, USA).

#### PET data preprocessing

Data were motion-corrected using frame-to-frame realignment and spatially aligned with the individual’s T1-weighted structural 3D images. To correct for head movement during the scan, nonattenuation corrected dynamic images were denoised using a level 2, order 64 Battle-Lemarie wavelet filter^49^, and individual frames were realigned to a single frame acquired four minutes after [^11^C]MePPEP injection using a mutual information algorithm^50^. The transformation parameters were then applied to the corresponding attenuation-corrected frames, and the realigned frames were combined to create a movement-corrected dynamic image (from 1.5 to 95 minutes following [^11^C]MePPEP administration) for analysis. The ADD images, created by weighted sum of the radioactivity images during the whole scan, of both the original and movement-corrected dynamic PET scans were used to create binary masks encompassing ~10 mm around the cortical surface as well as both grey and white matter^47^.

#### PET data kinetic analyses

[^11^C]MePPEP volume-of-distribution (V_T_) (which is the ratio at equilibrium of radioligand concentration in tissue relative to that in plasma^51^) was quantified applying a model-free analysis method (spectral analysis with rank shaping regularisation) previously validated for this radioligand^40^. [^11^C]MePPEP V_T_, which is proportional to CB1 receptor density and shows good test-retest reliability^18,40^ was calculated in the amygdala using an arterial input function and spectral analysis with rank shaping regularization (orthogonalized-functional-base)^52^ using in-house “Clickfit” software. The dynamic PET images within the binary masks and the metabolite-corrected arterial plasma input function were used to compute [^11^C]MePPEP V_T_ maps and the input response function at 60 min using spectral analysis^53^ implemented with the non-negative least squares algorithm^53^ within RPM software (available via http://www.bic.mni.mcgill.ca/~rgunn/Software.html). V_T_ refers to the equilibrium ratio of radioactivity in tissue compared to plasma and is proportional to the availability of the receptor or its density. For spectral analysis, the fast frequency boundary was kept at 0.1 s^−1^ throughout. The slowest kinetics should correspond to the physical decay of the isotope. In the case of [^11^C]MePPEP, the half-life of 20.4 min for^11^C gives a decay constant of 0.0005663 s^−1^, the log_10_ of which is −3.25. Here the slow frequency boundary chosen was 0.00063 s^−1^, relating to the log_10_ value of −3.2. The weights *w*_*i*_ for the individual data points were defined proportional to the reciprocal of the variance which was estimated from the scanner’s rate of true coincidences *T* (in s^−1^) as

$${w}_{i}=Li/Ti\,(forframei=1,\,2,\,3,\,\ldots .)$$

with *L* as the length of the frame (in seconds)^54^.

Tracer arrival delay was calculated with a basis functions method using the parent radiotracer input function and the tissue data information in the form of true coincidence detection and frame duration^48^ and was fixed for the entire brain. The contribution of the activity in the vasculature to the tissue response function (“blood volume”) was allowed to vary.

Anatomical parcellation of V_T_ maps was performed using a fully manually constructed, label-based maximum probability atlas designed for young people as studied here^55^. Volumetric results obtained using this atlas have been found to be comparable across studies for temporal lobe structures including the amygdalae^55^. A [^11^C]MePPEP template was normalized together with the volume of interest map to each individual PET ADD image using the statistical parametric mapping suite SPM5 (http://fil.ion.ucl.ac.uk/spm). In the present study, V_T_ was quantified in the amygdala due to its central role in emotional processing^11^ and the specific effects of delta-9-THC on emotional processing in the amygdala^3,13^.

### Statistical analysis

#### Behavioural data

Analyses of behavioural data were performed in SPSS version 21. For each psychopathological measure, the area under the curve (AUC) of effects versus time was estimated by the trapezoidal rule. Non-parametric approaches (Friedman Test) were employed for the analysis of psychopathological measures because of absence of variance during the placebo conditions and skewed responses during the delta-9-THC conditions. The change from baseline was assessed for normality employing the Kolmogorov-Smirnov test. The overall alpha level for each hypothesis was fixed at 0.05. Correction for multiple testing was applied (when relevant) within but not across the hypotheses, when relevant. Measures of task performance for the fMRI experiments were analysed using repeated measures ANOVAs used to compare drug conditions.

#### Correlational analyses

The hypothesized relationship between amygdala V_T_ values and anxiety symptoms was examined using Pearson’s product-moment correlation coefficient in SPSS version 21. To test the specificity of the relationship between anxiety symptoms and amygdala V_T_ values, this analysis was repeated post-hoc with the effect of delta-9-THC on the PANSS positive subscale score. As the fMRI data were acquired between one and two hours following drug administration, mean symptom ratings between one and two hours after drug administration was the measure employed for the purpose of correlational analyses with the symptomatic effects of delta-9-THC. As the result of our whole brain fMRI analysis revealed a significant acute effect of delta-9-THC on the right amygdala during the processing of fearful facial stimuli, we tested the relationship between CB1 receptor availability and the effects of delta-9-THC on anxiety and neural function using CB1 measures (V_T_) in the right amygdala.

First, we extracted the mean SSQ value from the amygdala cluster significantly modulated by delta-9-THC relative to the placebo condition for the fMRI contrast (fearful relative to neutral faces- this was localized to the right amygdala). We then estimated the Pearson’s product-moment correlation coefficient between these SSQ values and right amygdala V_T_ values in SPSS. The statistical significance threshold was set as *p* < 0.05.

All data generated or analysed during this study are included in this published article.

## Results

### Effects of delta-9-THC on drug levels and symptoms

**Figure 2:**
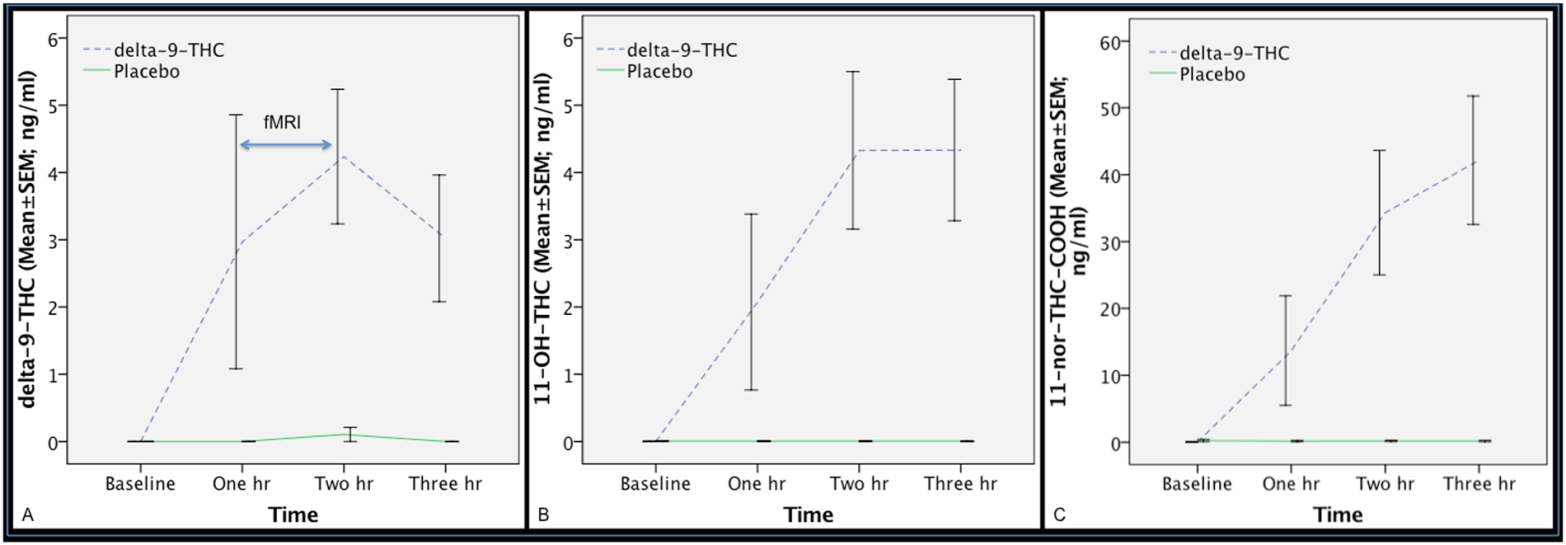
Blood levels of delta-9-THC (**A**) and its metabolites 11-OH-delta-9-THC (**B**) and 11-nor-9-carboxy-delta-9-THC (**C**), following the administration of delta-9-THC (interrupted blue line) or placebo (continuous green line). fMRI was acquired between 1 and 2 hour time-point.

Blood levels of delta-9-THC and its metabolites 11-OH-delta-9-THC and 11-nor-9-carboxy-delta-9-THC (Fig. 2A–C) were raised following delta-9-THC administration compared to the placebo condition and were not correlated with symptomatic and neural effects as delta-9-THC levels are out of phase (hysteresis) with its CNS behavioural effects^56^.

**Figure 3:**
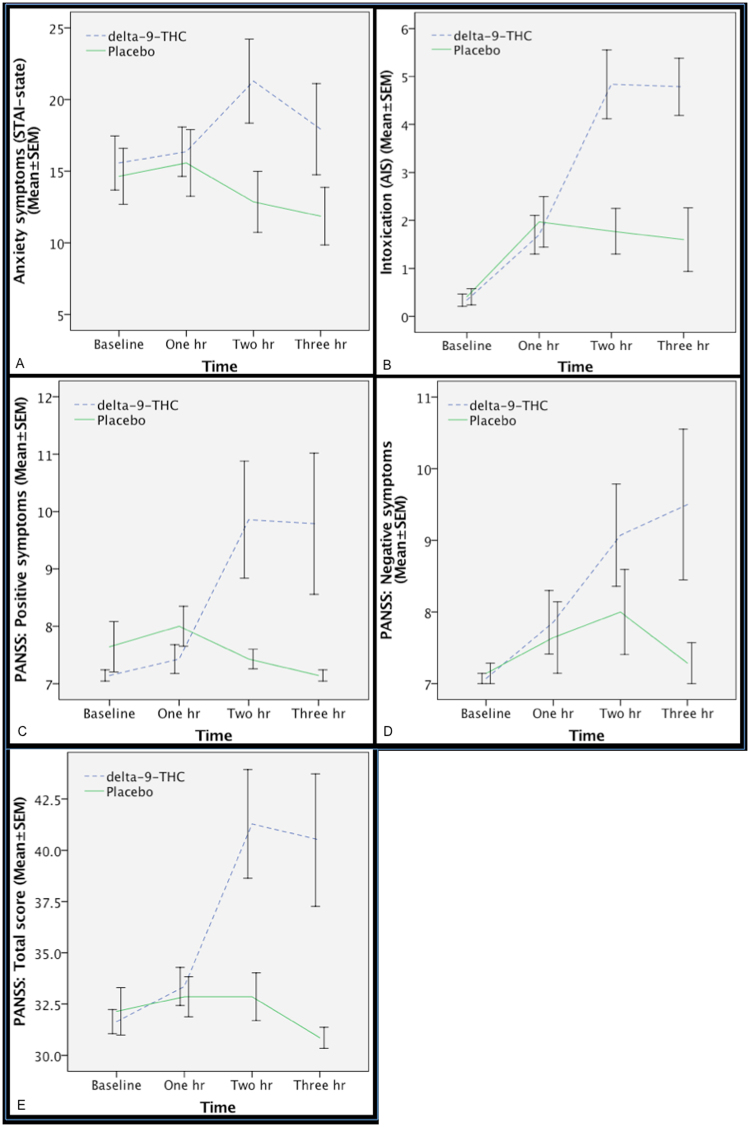
Effect of delta-9-THC (interrupted blue line) and placebo (continuous green line) on anxiety (χ^2^ = 7.14, *p* = 0.008; Fig. 2A), intoxication (χ^2^ = 10.28, *p* = 0.001; Fig. 2B), PANSS positive (χ^2^ = 4.45, *p* = 0.035; Fig. 2C), PANSS negative (χ^2^ = 6.23, *p* = 0.013; Fig. 2D) and total PANSS ratings (χ^2^ = 10.28, *p* = 0.001; Fig. 2E).

As expected, delta-9-THC transiently increased subjective ratings of anxiety, and intoxication as well as ratings on positive and negative PANSS subscales and total PANSS score (Fig. 3A–E) relative to the placebo condition.

### Effect of delta-9-THC on electrodermal response

**Figure 4:**
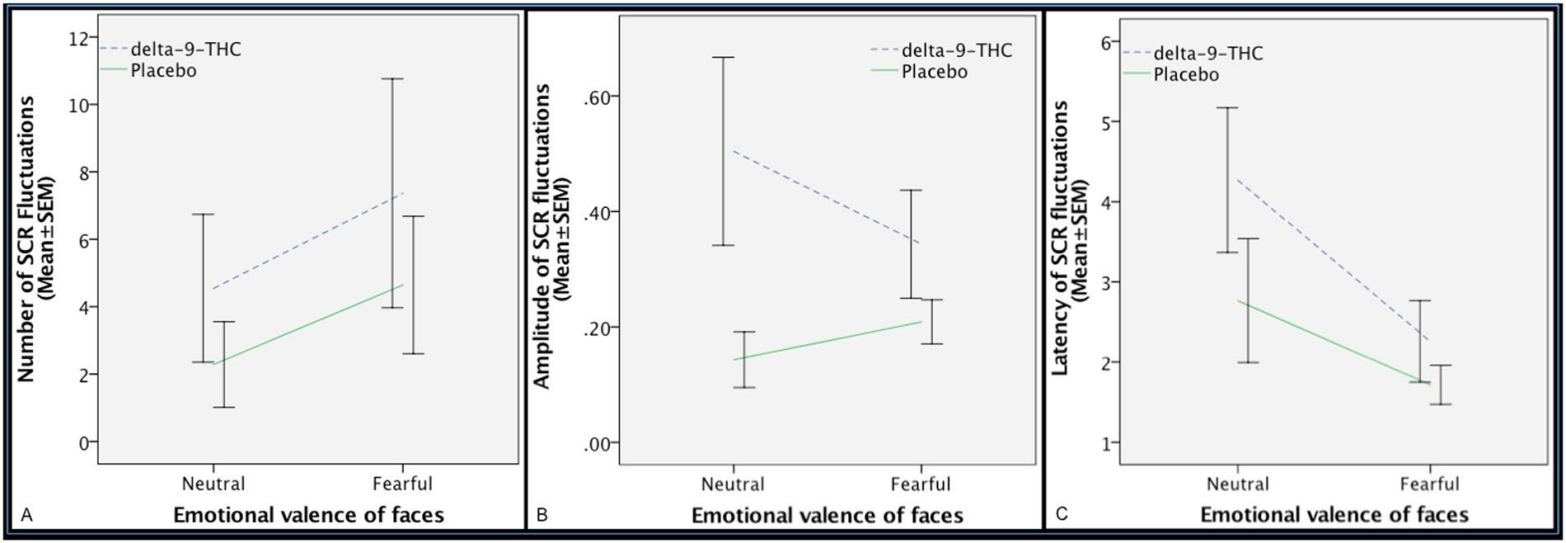
Effect of delta-9-THC (interrupted blue line) and placebo (continuous green line) on the number of fluctuations (**A**), amplitude (**B**) and latency (**C**) of skin conductance response (SCR) during the different emotional valence conditions of the facial stimuli shown during fMRI. Significant effect of emotional valence (neutral vs fearful) (F = 10.69, d.f. = 1, *p* = 0.003) on the number of SCR fluctuations. No significant effect of drug on SCR fluctuations.

There was a significant effect of emotional valence (neutral vs fearful) on the number of SCR fluctuations, such that the number of fluctuations was greater while viewing fearful faces as opposed to neutral faces across the drug conditions, indicating that the fearful stimuli were able to induce anxiety. Although the number of SCR fluctuations was greater under the influence of delta-9-THC relative to the placebo condition, this was not statistically significant. Nor was there any significant effect of drug by emotional valence interaction on the number of SCR fluctuations (Fig. 4A–C). There was no significant main effect of emotional valence (neutral vs fearful) (p = 0.62) on the amplitude (Fig. 4B) of SCR fluctuations. There was a significant main effect of drug treatment (F = 6.64, d.f. = 1, p = 0.014) on the amplitude of SCR such that SCR amplitude was greater under delta-9-THC relative to placebo for all faces stimuli. Although, there was no significant effect of interaction (*p* = 0.24) between drug treatment and emotional valence on SCR amplitude, the effect of emotional valence on SCR amplitude was in opposite direction in the two drug conditions, such that while the amplitude of SCR was greater while viewing fearful faces as opposed to neutral faces under the placebo condition, this was opposite direction under the influence of delta-9-THC.

There was a significant main effect of emotional valence (neutral vs fearful) (F = 6.90, d.f. = 1, p = 0.013) on the latency of SCR fluctuations, such that the latency was shorter while viewing fearful faces as opposed to neutral faces across the drug conditions (Fig. 4C). There was a trend-level effect of drug treatment (F_*1,35*_ = 3.07, p = 0.08) on the latency of SCR fluctuations such that SCR latency was greater under delta-9-THC relative to placebo across the neutral and fearful faces stimuli. However, there was no significant effect of interaction (*p* = 0.41) between drug treatment and emotional valence on SCR latency.

### Effect of delta-9-THC on fMRI task performance

Gender identification performance was comparable with previous studies^24^ and there was no significant main effect of emotional valence or drug treatment or an interaction between emotional valence and drug treatment on the ability of participants to distinguish male and female faces.

**Figure 5:**
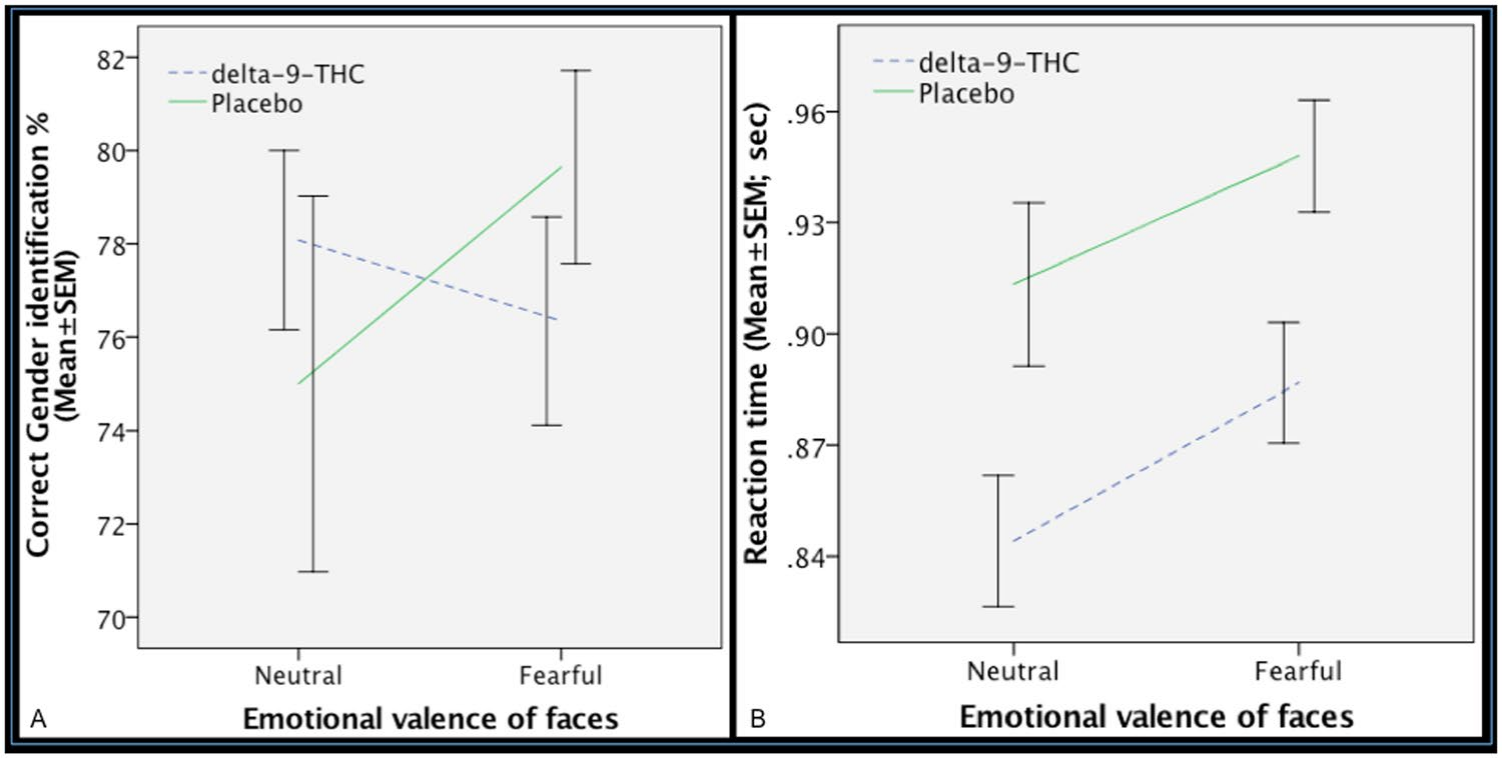
Effect of delta-9-THC (interrupted blue line) and placebo (continuous green line) on gender identification performance as indexed by the percentage of correct gender identification (**A**) and reaction time (**B**) while implicitly processing facial stimuli during the acquisition of fMRI. No significant main effect of emotional valence (*p* = 0.59) or drug treatment (*p* = 0.96) or an interaction (*p* = 0.23) between emotional valence and drug treatment on the ability of participants to distinguish male and female faces. Significant effect of emotional valence (neutral vs fearful) (F = 4.32, d.f. = 1, *p* = 0.038) and drug condition (F = 12.30, d.f. = 1, *p* < 0.001) on reaction time. No significant effect of interaction (F = 0.04, d.f. = 1, *p* = 0.829) between emotional valence and drug condition on response latency.

There was a significant effect of emotional valence (neutral vs fearful) and drug condition on reaction time, such that response latency was greater for fearful as opposed to neutral faces across both drug conditions and shorter under the delta-9-THC condition relative to the placebo condition across all emotional valence conditions. However, there was no significant effect of interaction between emotional valence and drug condition on response latency (Fig. 5A–,B).

### Effect of delta-9-THC on neural activation

**Figure 6:**
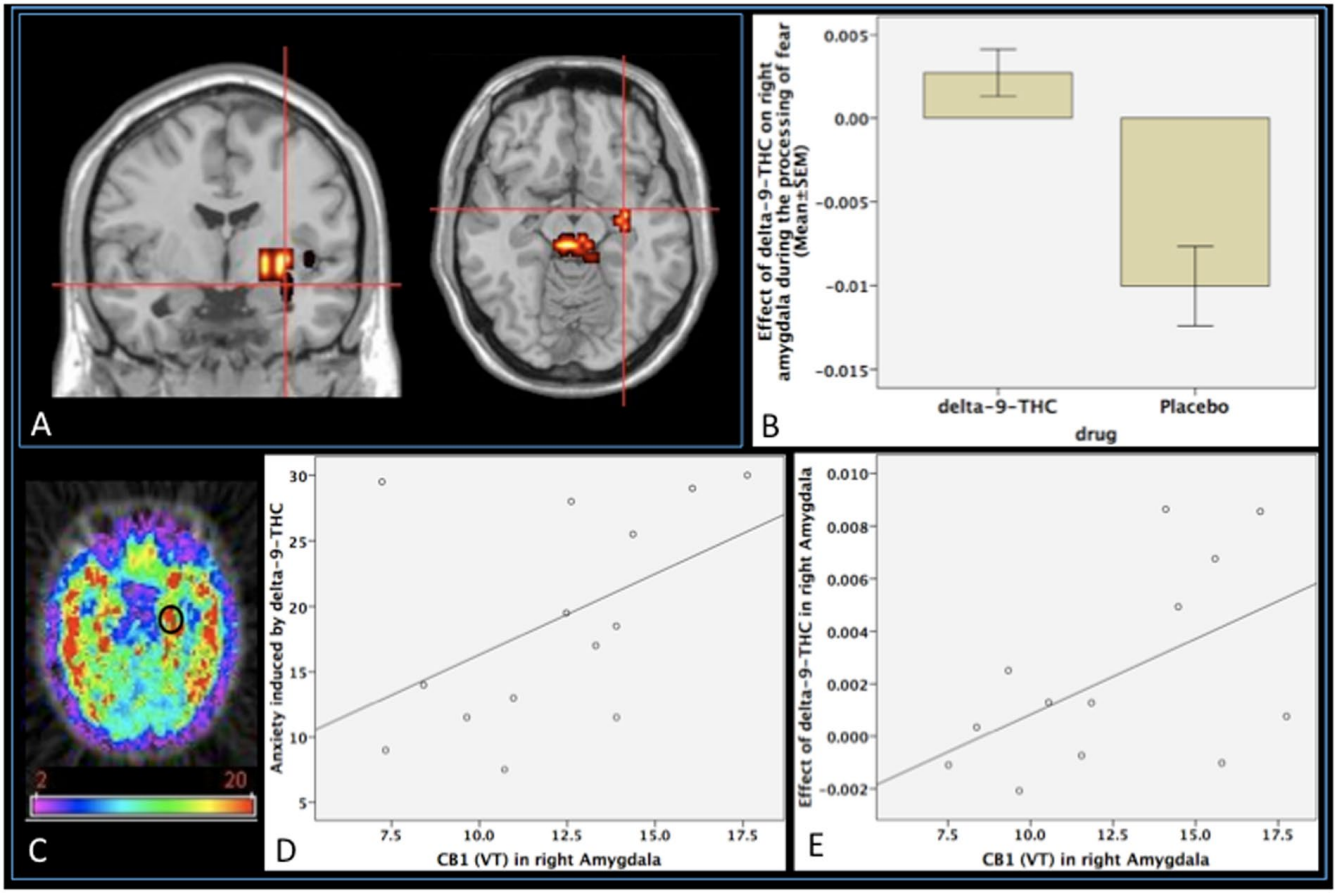
(**A**) Effect of delta-9-THC on activation relative to placebo in the right amygdala (cross-hairs) while subjects viewed fearful relative to neutral facial stimuli (*p* = 0.0004, corrected for <1 false-positive cluster; peak focus x = 29, y = 4, z = −13, coordinates in Talairach space). (**B**) Plot showing the mean magnitude of activation (indexed by the sum of squares ratio, y-axis; error bars show standard error of the mean) in the right amygdala following administration of delta-9-THC and placebo, respectively. (**C**) Image showing CB1 receptor availability in the amygdala (encircled) assessed using [^11^C]MePPEP PET [Mean (SD) CB1 V_T_ in right amygdala: 12.57 (3.27) ml/g; Range: 7.51–17.75 ml/g]. (**D**) Correlation between the anxiety symptoms induced by delta-9-THC and CB1 V_T_ in the right amygdala. (**E**) Direct correlation between the effect of delta-9-THC on the right amygdalar response while subjects viewed fearful faces and CB1 V_T_ in the right amygdala.

Administration of delta-9-THC was associated with the modulation of activation, as indexed by fMRI BOLD response, in a network of brain regions that included the right amygdala extending to the hippocampus as well as the midbrain, striatum, insula and thalamus during the processing of fearful faces relative to the neutral faces condition (Fig. 6A and B; Table 2). This region plays a critical role in fear processing and has previously been implicated in mediating the effects of delta-9-THC in man^3,13^. As shown in Fig. 6B, under placebo conditions, there was a greater response to neutral than to fearful faces in the right amygdala. However, following administration of delta-9-THC, the opposite applied, with a greater response to fearful than to neutral faces. Enhancement of the BOLD response in the right amygdala under delta-9-THC significantly was significantly related to its effect on the number of SCR fluctuations while processing fearful faces (Pearson’s r = 0.67, *p* = 0.012), suggesting that the effects of delta-9-THC on two different physiological measures concomitantly obtained while processing fearful stimuli were directly related.

**Table 2:** Brain regions modulated by delta-9-THC during fear processing (*p* = 0.0004, corrected for <1 false-positive cluster).

Cluster Size	Talairach coordinates			Hemisphere	Region
	X	y	z		
27	29	−4	−13	R	Amygdala
9	36	−33	4	R	Hippocampus
72	22	11	−7	R	Striatum
16	40	−11	−2	R	Insula
11	22	−19	4	R	Thalamus
22	7	−19	−13	R	Midbrain

### Relationship between CB1 receptor availability and effects of delta-9-THC on symptoms and neural function

There was a positive correlation between the anxiogenic effect of delta-9-THC and CB1 receptor V_T_ in the right amygdala (r = 0.47, p = 0.04; Fig. 6D), with greater CB1 receptor V_T_ associated with a greater severity of delta-9-THC - induced anxiety. Furthermore, post-hoc testing revealed that there was no significant relationship between amygdala CB1 receptor V_T_ and the severity of transient psychotic symptoms induced by delta-9-THC, indicating that this relationship was specific to anxiety symptoms.

Similarly, there was a significant positive relationship between the effect of delta-9-THC on activation in the right amygdala during fear processing (Fig. 6A and B) and CB1 receptor V_T_ in the right amygdala (Fig. 6C): greater CB1 receptor V_T_ was associated with a greater effect of delta-9-THC on the amygdalar response (r = 0.53, p = 0.03; Fig. 6E). Post-hoc analyses indicated that this relationship with CB1 receptor V_T_ was region-specific: it was not evident in other regions where the task response was modulated by delta-9-THC, such as the insula and midbrain.

## Discussion

Employing a multimodal imaging approach that involved estimation of baseline receptor availability using PET imaging and estimation of effects of delta-9-THC on neural function and behavior using a combination of pharmacological challenge and fMRI, we investigated a group of healthy volunteers with minimal previous exposure to cannabis. The principal findings of this study are that a modest dose of delta-9-THC resulted in the acute induction of anxiety symptoms in the healthy volunteers studied here and the severity of anxiety induced by delta-9-THC was directly correlated with the baseline availability of CB1 receptors in the amygdala, a region that has been linked to anxiety and fear processing both in health^11^, and under the influence of delta-9-THC^3^. Furthermore, these symptomatic effects of delta-9-THC were associated with enhanced engagement of the amygdala and adjoining hippocampus during the processing of fearful facial stimuli and this effect of delta-9-THC on amygdala activation was directly correlated with the availability of CB1 receptors in the same region. Although the amygdala has previously been implicated as the substrate mediating the effects of delta-9-THC on emotion in man^3,13^, the precise nature of these effects and their molecular underpinnings were unclear. This is the first evidence that the effects of delta-9-THC on anxiety and on the physiological response of amygdala, a key neural substrate for anxiety during the automatic processing of fearful stimuli in humans are related to the local availability of CB1 receptors and is consistent with independent recent evidence suggesting greater CB1 receptor availability throughout the brain including amygdala in patients with post-traumatic stress disorder (PTSD) relative to healthy controls^57^ and an association between amygdala CB1 receptor availability and attentional bias to threat and threat-related symptoms in those presenting with trauma-related psychopathology^58^. It is also consistent with evidence that direct administration of delta-9-THC to the amygdala in rats induces fearful behaviors, whilst CB1 receptor blockade using an inverse agonist has the opposite effect^9^. Moreover, this relationship between the effects of delta-9-THC and the availability of CB1 receptors appeared to be both region- and symptom-specific. It was not evident in other brain areas, in line with reports that in rodents, application of delta-9-THC to the amygdala, but not the prefrontal cortex or hippocampus, induces fearful behaviors^9^. Similarly, neither the response to fearful faces nor CB1 availability in the amygdala were related to other phenomena induced by delta-9-THC, such as psychotic symptoms, consistent with evidence that application of delta-9-THC to the amygdala in rats is associated with fearful behaviours, but not hyperlocomotion^9^, a feature of animal models of psychosis. However, it is worth noting that within individual binding of [^11^C]MePPEP is strongly correlated between various brain regions. Hence, it is possible that the association observed between amygdala CB1 receptor availability and the effects of delta-9-THC may be secondary to association with whole brain CB1 receptor availability rather than reflecting a specific association with the amygdala, which merits investigation in future studies with larger samples.

Acute and transient symptoms similar to that induced by delta-9-THC in the present study have also been reported in the context of recreational cannabis use^2,59^ and in experimental studies involving the administration of delta-9-THC^26^ in healthy human volunteers. However, doses of orally administered delta-9-THC lower than that used here, have been associated with either a reduction in anxiety or no clear anxiogenic/relaxing effect^13,25^. It is well known that there is a great deal of variability in the effects of delta-9-THC or cannabis in a given individual. While a number of different factors, in particular genetically mediated differential sensitivity^22^, dose of delta-9-THC^2,3,13,25^, and the presence of other cannabinoids such as Cannabidiol^3^, are likely to contribute to this variability between different individuals in their response to cannabis, results of the present study suggest that baseline availability of CB1 receptors, the central molecular target of delta-9-THC, may also partly explain differential sensitivity to the anxiety experienced under the influence of cannabis.

While making a gender decision during the event-related implicit fear processing fMRI task, participants performed comparably across the two drug treatments as well as emotional valence conditions, suggesting that they were attending to the task stimuli across all conditions. Across drug conditions, response latency was faster for neutral faces relative to fearful faces consistent with previous studies employing negative facial stimuli^13,60^. Shorter response latency to all facial stimuli while making a gender decision under delta-9-THC is consistent with evidence of faster responding under its influence^22^. Consistent with previous studies^13,60^, there was no significant interaction between drug treatment and emotional valence on response latency. Hence, any effects of delta-9-THC on neural (BOLD response) or electrodermal (SCR) measures are unlikely to be related to its effect on performance or attention during the task. Viewing fearful faces was associated with increase in SCR fluctuations suggesting that the fearful faces were indeed associated with changes in measures of autonomic arousal characteristic of anxiety^23,61^. Effect of delta-9-THC on amygdala engagement while processing fearful stimuli were directly related to its effect on a concomitantly recorded arousal measure (SCR fluctuations) linked to the presentation of fearful stimuli. Enhanced engagement of amygdala while processing fearful faces relative to neutral faces under the influence of delta-9-THC as noted in the present study is however different from its attenuating effect on amygdala reactivity during the processing of a different set of negative emotional stimuli as previously reported by Phan *et al*.^13^. Use of a lower, and hence potentially anxiolytic dose of delta-9-THC^62^ as well as an explicit emotion processing task [linked to attenuation of amygdala activity] employed by Phan and colleagues, unlike the present study may partly explain this. Additionally, they^13^ compared threatening (angry/fearful) with non-threatening (happy) facial stimuli, which may have resulted in a greater effect of the lower dose of delta-9-THC during the control condition (happy faces) compared to the active (angry/fearful faces) condition, thereby resulting in the attenuation of amygdala response. Consistent with this, they also reported that amygdala reactivity to happy faces was greater under the influence of delta-9-THC relative to the placebo condition when they employed a different control task (matching geometric shapes), which was interpreted as indicative of the pro-social effects of delta-9-THC, unlike the anxiogenic dose employed in the present study.

It is worth noting that in the present study there was greater amygdala engagement in response to neutral faces relative to fearful faces under placebo condition. Engagement of amygdala whilst making a gender decision in response to neutral faces is consistent with a prominent role for amygdala in adaptive social behaviours such as face perception and evaluation^63^. While the relatively lesser amygdala engagement to fearful faces under placebo condition may appear counterintuitive, it is perhaps not entirely unexpected, as the probability of amygdala activation is lower whilst performing emotion-processing tasks requiring additional effort, such as making a gender decision^11,64^ linked to inhibitory effect of prefrontal and anterior cingulate cortex on amygdala function^65^.

Another important aspect of the present results relate to the right lateralization of the neural effects of delta-9-THC. While not predicted *a priori*, it is consistent with a previous study^13^ and in accordance with current understanding regarding hemispheric lateralization of emotional processing with unconscious processing of emotional stimuli (as required in the present study) in the right amygdala and a more conscious processing of emotion in the left amygdala^66^ and evidence from meta-analyses of neuroimaging studies of emotional processing^11,67^. Lateralization of emotional processing has been shown to be more prominent in men than in women^67^ and the present study involved only male participants. Another issue that is worth noting in this context relates to our previous cannabinoid challenge fMRI study^3^, where we observed net activation in the left amygdala under the placebo condition using the same fear processing task in contrast to the net deactivation in right amygdala under placebo condition observed in the present study. In the previous study our analysis aimed to selectively identify brain areas where the effects of delta-9-THC and Cannabidiol were in the opposite direction relative to the placebo condition for each task condition studied there, including the fear processing task. Hence, it is very likely that difference in left amygdala activation between the drug conditions observed in that study was driven by the effect of Cannabidiol, which was observed in the left amygdala when it was contrasted directly with the placebo condition, as we have also reported in a related study investigating the same dataset^23^. In contrast, in the present study, we have directly compared the effects of delta-9-THC and placebo (as opposed to carrying out a 3-condition analysis) in a separate group of healthy volunteers whilst processing fearful compared to neutral faces. This may underlie the difference in lateralization of fear processing observed under the placebo condition in the two studies, driven by differences in the brain effects of Cannabidiol, an anxiolytic and delta-9-THC, having anxiogenic effects. The difference in the direction of effects in the two amygdalae under placebo may reflect greater right amygdala activity in response to neutral faces^68^ as well as faster habituation of right amygdala activity compared to the left^68,69^ and is consistent with evidence suggesting that while the left amygdala functions as a specific detector^70^ and sustained evaluator^69^ of fearful faces, the right amygdala functions more as a non-specific detector of change initiating a rapid, automatic but general level of response to all types of facial stimuli (whether neutral or fearful)^70,71^. To summarize, while the amygdala lateralization difference between the two studies using the same fMRI paradigm are likely a result of differences between the effects of CBD and delta-9-THC (which have opposing effects on anxiety), differences in the net effects in the amygdalae under placebo condition in these two studies likely reflect inherent differences in their role in the processing of fearful and other facial stimuli.

However, the results of the present study need to considered together with certain caveats. It is particularly important to note that the interval between PET and fMRI scan acquisition was 25.3 ± 16.9 months. Multi-modal imaging studies such this, especially those that also involve pharmacological challenge are logistically complex. The rather long delay between the two types of imaging in the present study was also a result of delays in securing funding, obtaining necessary regulatory approval and delays in the production of the PET tracer that passed quality control as well as in securing PET scanning slots. As the stability/variability of CB1 receptor levels in man over such long periods is unclear, arguably, this may be a particular concern given the relatively high test-retest variability of [^11^C]MePPEP^72^. For example, using rank-shaping regularization of spectral analysis, the median test-retest variability of [^11^C]MePPEP has been reported to be at least 14% (ranging from −48.5 to 34.9%) when scans were repeated a median of 24 days apart^72^. Other work has also pointed towards high between-subject variability of^11^C]MePPEP^17^. However, it is worth noting that any such variability is more likely than not to have resulted in increased noise and thereby weakened the association between CB1 receptor V_T_ and the effects of delta-9-THC on symptoms (anxiety) and brain activation (amygdala BOLD signal). Furthermore, variability in CB1 receptor V_T_ as measured using PET may not necessarily reflect variability in CB1 receptor density over time, but may also be related to the specific tracer. The test-retest variability of the volume of distribution (V_T_) of [^11^C] ligands measured with PET in humans with current scanning technology varies between 6% in medium to large cortical areas, for well characterized tracers, to 12% in less well characterized ones^73–75^. Hence, median variability of 14% as in the case of [^11^C]MePPEP is particularly at the higher end for PET radioligands. However, the intra-class correlation coefficient on [^11^C]MePPEP data analyzed with some form of spectral analysis, and rank shaping in particular that is better able to control the variance given the very slow kinetic of the tracer, on average is greater than 0.8, which makes [^11^C]MePPEP a very sensitive/accurate PET ligand perfectly suitable for the experimental design that we have employed here. In light of above, whilst it is very unlikely that the interval between fMRI and PET systematically affected the direction of our results, we cannot completely rule out this possibility. Hence, future studies may need to employ simultaneous PET-MR designs to acquire such data.

A further consideration while interpreting these results relate to challenges in accurately delineating the amygdala despite the use of label-based maximum probability atlas that we have employed in this study for the anatomic parcellation of V_T_ maps. This is further compounded by limitations imposed by MRI scanner resoultion particularly on the delineation of smaller structures such as the amygdala. However, it is worth noting that while these effects may have affected absolute CB1 quantification in the amygdala, this would have affected all participants in an identical manner rather than introduced any systematic bias and hence is unlikely to have affected the direction of the association between CB1 V_T_ and effects of delta-9-THC on anxiety and the BOLD signal reported here. It also worth noting that we did not correct the results of our correlational analyses for multiple comparisons. However, it is important to note that we had two specific hypotheses based on previous literature regarding the relationship between CB1 availability and effects of delta-9-THC on anxiety and amygdala activation, which we tested in our correlational analyses, focusing specifically on the right amygdala as that is where the acute effect of delta-9-THC was observed using fMRI whilst processing fearful faces. As these were hypothesis-driven correlational analyses and not strictly independent because they examine the relationship between CB1 V_T_ in the same brain region (amygdala) with two different effects produced by the same intervention (effect of delta-9-THC on brain activation and anxiety), we have not corrected them nor the post-hoc correlational analyses for multiple comparisons.

While one may question the generalizability of these results as street cannabis has many different ingredients, unlike pure delta-9-THC administered here, it must be noted that delta-9-THC produces subjective effects similar to that of an extract of the cannabis plant^76^. Generalizability of these results in occasional cannabis users to regular cannabis users may also be questioned in light of evidence of downregulation of CB1 receptors across several cortical regions in chronic cannabis users relative to control subjects^77^. While alteration of CB1 receptor availability is definitely likely to affect the acute behavioural and neural response to cannabis in regular users, results reported here provide preliminary evidence that, if replicated in independent samples, may explain variable sensitivity to the adverse effects experienced by regular users when they start experimenting with cannabis, as well as the huge majority of individuals who only use it occasionally and never go on to use regularly. Furthermore, one needs to be careful in terms of generalizing these results to those with anxiety disorders, as cannabis is often used in an attempt to seek relief from anxiety, unlike the relationship with anxiogenic effects tested here. It is important to also recognize that we tested only males and gender difference in the effects of cannabinoids is well-recognized^78^ and have also been demonstrated recently with regard to CB1 availability in both healthy individuals and those with PTSD^57^. Another caveat relates to the use of V_T_ to quantify binding of [^11^C]MePPEP to CB1 receptors, as it incorporates both specific as well as non-displaceable binding. While directly estimating binding potential (BP_ND_), a measure of specific binding would have been ideal, this has not been possible for CB1 receptors to date^58,72,79^ owing to lack of a brain region devoid of CB1 receptors that may be suitable for use as a reference region. A previous attempt to use the Pons, a region with relatively low CB1 receptor concentration^14,15^ as a pseudo-reference region yielded inconsistent and unreliable results^17^. Hence, the most definitive way to address this issue would entail a blocking study in man using a selective CB1 receptor antagonist. However, such data are currently unavailable owing to lack of availability of a selective antagonist suitable for human experimentation. Animal studies however suggest high specific binding with values of over 85% in the monkey brain^14–16,18^.

Notwithstanding these caveats, the present multimodal imaging study firstly demonstrates that a single modest dose of delta-9-THC induced transient anxiety symptoms in healthy volunteers with minimal previous expsoure to cannabis and that this effect was associated with its effect on two concomitantly acquired physiological parameters linked to autonomic arousal and anxiety measured while processing fearful stimuli, fluctuations in skin conductance and amygdala BOLD response as evident using a robust whole-brain image analysis approach, which themselves correlated with each other. Finally, this study links both the symptomatic and neural effects of delta-9-THC to the baseline availability of its main central molecular target. Together, these findings suggest that the anxiogenic effects of cannabis in humans are mediated by CB1 receptors in the amygdala. This raises the possibility that the endogenous cannabinoids that normally act on these receptors play a role in modulating anxiety, independent of cannabis use. The endocannabinoid system thus represents a logical pharmacological target for the development of novel treatments for anxiety disorders.
